# Early Response QTLs and DEGs Underlying Oil Palm Resistance to *Ganoderma boninense* in Two Breeding Populations Evaluated in Pre-Nursery Trials

**DOI:** 10.3390/microorganisms14081712

**Published:** 2026-08-04

**Authors:** Aurélie Daval, David Lopez, Teresa Cuellar, Aqdi Prasetio, Marie Denis, Alexandre Soriano, Solène Puerto, Dadang Afandi, Virginie Pomiès, Jeanne Danon, Deni Arifiyanto, Camille Madec, Younes Amara, Virginie Riou, Indra Syahputra, Florence Jacob, Norbert Billotte, Sébastien Tisné

**Affiliations:** 1 CIRAD, UMR AGAP Institute, 34398 Montpellier, France; aurelie.daval@cirad.fr (A.D.); david.lopez@cirad.fr (D.L.); teresa.cuellar@cirad.fr (T.C.); marie.denis@cirad.fr (M.D.); alexandre.soriano@cirad.fr (A.S.); solene.puerto@cirad.fr (S.P.); virginie.pomies@cirad.fr (V.P.); virginie.riou@cirad.fr (V.R.); norbert.billotte@cirad.fr (N.B.); 2 UMR AGAP Institute, University Montpellier, CIRAD, INRAE, Institute Agro, 34398 Montpellier, France; 3 P.T SOCFINDO, Jl. Yos Sudarso, Medan 20115, Indonesia; aqdip@socfindo.co.id (A.P.); dadanga@socfindo.co.id (D.A.); denia@socfindo.co.id (D.A.); indras@socfindo.co.id (I.S.); 4 PalmElit SAS, 34980 Montferrier-sur-Lez, France; camille.madec@palmelit.com (C.M.); younes.amara@palmelit.com (Y.A.); florence.jacob@palmelit.com (F.J.); 5 UMR GDEC, INRAE-UCA, 63000 Clermont-Ferrand, France; jeanne.danon@inrae.fr

**Keywords:** *Elaeis guineensis*, basal stem rot disease, QTL mapping, RNA sequencing, Bayesian variable selection, marker-assisted selection

## Abstract

*Ganoderma boninense*, a soil-borne pathogenic fungus causing basal stem rot (BSR) disease in oil palm, is the major threat to cultivation in Southeast Asia, with the potential to reduce oil production by up to 80% in severely affected plantations. With a view to marker-assisted selection (MAS) of resistant planting material, we combined genetics and transcriptomics approaches to investigate the defense mechanisms of oil palm against infection by *G. boninense*. We first performed quantitative trait locus (QTL) mapping of BSR resistance using a multi-locus Bayesian variable selection approach applied to more than 10 years of data collected during pre-nursery tests of Deli and La Mé breeding populations. We found 6 and 5 BSR resistance loci segregating in Deli and La Mé respectively, with no overlap between them. Then we performed an RNA-sequencing approach on libraries of roots and bole of oil palm seedlings inoculated or not with *G. boninense*, focusing on Deli × La Mé crosses with contrasted BSR resistance. A Bayesian variable selection on predictors obtained by tracing the resistance loci haplotypes in profiled individuals identified 195 differentially expressed genes (DEGs) for the *G. boninense* inoculation × QTL interaction, among the 551 DEGs found for inoculation effects. The DEGs were located both in *cis* and *trans* positions compared to the segregating QTL intervals, enabling the prioritization of candidate genes and resistance mechanisms associated. Focusing on a strong effect QTL region on chromosome 4 in the Deli genetic background, we identified three candidate genes in *cis* positions that exhibited differential expression both in response to *G. boninense* inoculation and between resistant and susceptible haplotypes. Our study is the first combining QTL and RNA-seq approaches in oil palm based on genetically connected experimental setup, providing valuable information on the underlying mechanisms and paving the way for MAS of BSR resistant planting material.

## 1. Introduction

The oil palm (*Elaeis guineensis* Jacq.) is the leading edible oil-producing species within the Arecaceae family and the most productive oil crop worldwide. With a global annual production of around 90 Mt, oil palm accounts for 36% of the global vegetable oil produced on only 7% of the 300 M ha of land dedicated to vegetable oil production [1]. A strong concern for oil palm is about pests and diseases that threaten palm oil production in all areas of cultivation and contribute to the strong current yield gap [2]. Furthermore, studies including climate change scenarios indicate that this threat will worsen for the major oil palm diseases, namely Fusarium wilt and basal stem rot (BSR) caused by *Fusarium oxysporum* forma specialis (f. sp.) *elaeidis* and *Ganoderma* species respectively [3,4,5,6,7,8,9]. In Southeast Asia, BSR is caused by *Ganoderma boninense*, a soil-borne pathogenic basidiomycete that infects oil palms through their roots when they come into contact with infected debris or nearby diseased roots [10]. *G. boninense* can spread either locally via vegetative growth through root-to-root contact with dikaryotic mycelium, or over longer distances through sexual reproduction via monokaryotic basidiospores released from sporophores on the stems of infected trees [11,12,13,14]. For the latter, the fusion of two monokaryotic mycelia is required to form the dikaryotic mycelium, which represents the main infectious stage of the pathogen [11]. The effects of BSR on oil yield becomes significant when 10 to 20% of the oil palm trees are infected, while 30–70% of the trees may die over a typical 25-year planting cycle [15,16]. Hence, in this region producing 85–90% of global palm oil, replanting aging plantations without causing deforestation poses a major challenge, as reusing the same areas accelerates the spread of BSR due to the increase in inoculum load over repeated replanting cycles [17].

An integrated pest management approach including agricultural practices and resistant oil palm planting material would provide a sustainable response to this threat [18]. Oil palm breeders thus need to focus on developing resistant planting material, while maintaining or even improving oil yield. Despite steady genetic progress in palm oil yield and quality over the past century, most commercial oil palm planting material has undergone only a limited number of breeding generations due to the length of the selection cycle (15 to 20 years) [19]. Advances in genetics and genomics offer great potential for identifying the genetic determinants and molecular mechanisms underlying traits of interest by leveraging genetic variation, thereby improving the breeding process [20].

Most of the molecular research on BSR resistance published to date have focused on “-omics” studies with the aim of identifying the genes, proteins, and metabolic pathways affected by *G. boninense* infection: transcriptomic [21,22,23,24,25,26], proteomic [27,28] or metabolomic [29,30,31,32] approaches. Based on these results, a set of candidate genes and metabolites involved in BSR resistance has emerged, including genes involved in lignin biosynthesis and phenylpropanoid pathways [33,34] or defense-related genes in jasmonate and salicylate signaling pathways in relation with reactive oxygen species (ROS) response [35]. Zuhar et al. [26] validated the expression of ten defense- and secondary metabolism-related candidate genes in response to *G. boninense* infection in six-month-old oil palms, with seven of these genes also validated in 15-year-old trees. However, a comprehensive understanding is still lacking, as there is no methodological consensus for studying transcriptomes and candidate gene expression, particularly since the hemibiotrophic lifestyle of *G. boninense* triggers contrasting host responses across different infection stages [25]. Studying the genetic architecture of BSR resistance could help decipher this complex interaction and identify targets for breeding, but such studies remain scarce. An association between SSR alleles and BSR resistance was reported in three inoculated crosses [36], but the design and scope of the study does not allow for robust conclusions regarding the genetic architecture of BSR resistance. For species such as oil palm, which face significant biological constraints that limit the power and reliability of genetic analyses, the use of data from breeding programs offers considerable potential despite the methodological challenges involved [37]. Indeed, data are classically strongly unbalanced and noisy, and the complex pedigrees of breeding populations prevent the use of quantitative trait locus (QTL) detection methods designed for bi- or multi-parental (inbred) populations. Alternative approaches adapted to complex pedigrees have been proposed: frequentist methods using genomic relationship matrices based on molecular markers, known as “two-step variance component” [38] or “regional heritability mapping” [39] methods, or Bayesian approaches based on Identical By Descent (IBD) information and variable selection [40]. These methods applied to oil palm have enabled the identification of QTL for traits of interest based directly on data from breeding programs, such as yield components [41,42], BSR and Fusarium wilt resistance in early screening tests [43,44], and BSR resistance in the field [45].egarding resistance to *G. boninense*, these studies have highlighted its quantitative nature, supported by a polygenic architecture comprising numerous resistance loci specific to different genetic backgrounds and, occasionally, specific experimental conditions.

Given the diversity of experimental approaches and genetic material published to date, it seems impractical to combine the -omics and QTL results already available in the literature to define a clear direction for marker-assisted selection (MAS) of BSR resistance. To advance our understanding of the genetic and molecular architecture of BSR resistance, we conducted an integrated study using genetically related experimental designs to fully leverage QTL and transcriptomic approaches. Our work is based on standardized early screening tests that involve inoculating *G. boninense* in the pre-nursery stage [46,47] and on the central role of haplotype analyses in effectively combining both approaches. A flexible and powerful statistical framework using Bayesian variable selection implemented in the R package BGLR [48,49] was used to cope with the complexity of the experimental design and finally shed light on candidate genes and pathways underlying BSR resistance in two major populations for oil palm breeding.

## 2. Materials and Methods

### 2.1. QTL Mapping Approach

#### 2.1.1. Plant Material

The palm trees studied belong to the oil palm breeding program of CIRAD, its subsidiary PalmElit (Montferrier-sur-Lez, France) and their partner PT Socfin Indonesia (Medan, Indonesia). The breeding program was conducted in a recurrent reciprocal selection scheme with two heterotic groups A and B (GA and GB). The two heterotic groups have complementary yield component traits, with low fruit bunch numbers and high bunch weight in GA and reciprocally in GB. Parents from both heterotic groups were crossed to produce GA × GB hybrids showing a heterosis effect on fruit bunch yield and used as commercial planting material [50,51]. GA × GB hybrids make the most productive tenera fruit form, i.e., with a thin shell. Tenera are obtained when crossing individuals homozygote for the two alternative alleles of the SHELL gene, dura and pisifera [52].

The GA and GB parental populations studied for their BSR resistance consisted of individuals derived from the breeding populations of Deli (DE, Indonesia) and La Mé (LM, Ivory Coast) respectively. The current Deli population was established following several rounds of selection from four individuals planted in Indonesia in 1848 [53]. The GA/GB pedigrees studied comprised respectively 2347/450 individuals including founders, among them respectively 2069/361 parents were directly progeny-tested for BSR resistance in pre-nursery screening test and respectively 478/192 were genotyped (Figure S1). Finally, a total of 1092 GA × GB crosses for which both parents were genotyped were used for QTL mapping.

#### 2.1.2. Fungal Material

The *G. boninense* dikaryotic isolate used in this study (called NJ3) was collected in 2007 from the bole of an infected oil palm in Bangun Bandar PT Socfindo estate (North Sumatera, Indonesia). Then, it was purified and maintained on standard PDA medium (39 g·L^−1^ PDA, Sigma-Aldrich, St. Louis, MO, USA) added with 50 mg·L^−1^ chloramphenicol. The fungus cultures were incubated in the dark at 27 °C. Pathogenicity of NJ3 isolate was assessed on oil palm seedlings at Tanah Gambus PT Socfindo estate by using a standardized inoculation screening test set up [47]. Several dikaryotic clonal lines (CLs) were derived by sequentially recovering the isolate from the boles of young infected seedlings over successive pre-nursery trials. These host-passage steps prevented the loss of pathogenicity that frequently occurs during prolonged subculturing on artificial fungal media [54]. Genomic analyses of this isolate were previously conducted, providing a draft genome sequence and a characterization of its SSR markers [55]. To facilitate the harvesting of the mycelium for DNA sequencing, a cellulose sheet was deposited on the PDA medium surface prior to subculturing the fungus. NJ3 sequence data have been deposited in the NCBI, under the BioSample accession number SAMN54941916 (Bioprojects PRJNA1112779).

#### 2.1.3. Phenotypic Data

The pre-nursery screening tests were developed for the evaluation of the genetic resistance to BSR of breeding and commercial oil palm planting material, using controlled and standardized inoculation of germinated seeds [47]. A rubber wood block (RWB) colonized by *G. boninense* was used as an inoculum source, being installed in a nursery polybag before planting the germinated seed. NJ3 CLs (*n* = 7) were used in a series of trials conducted over a ten-year period, whose data were used for QTL mapping. A single pathogenic CL was used for all the crosses tested in a single trial. Around 100 crosses were evaluated simultaneously in each pre-nursery trial. Among them, 20% were control crosses from susceptible, intermediate and resistant genetic backgrounds, that were included in all the trials performed. Of the remaining 80% of crosses representing the tested crosses, 50% overlapped in two consecutive trials, leading to at least two independent testing per cross. At a trial level, each cross was represented by 100 inoculated germinated seeds clustered in five replicates following the protocol described by Breton et al. [46]. After the appearance of the first external disease symptom, the observation of the inoculated seedling switched from every four weeks to twice a month with a recording of (1) for infected and (0) if not infected, as described in [43]. The trial was stopped when the average percentage of infected seedlings within the group of control reached 30%, usually around 34 weeks after inoculation of the germinated seeds. This 30% threshold was determined to have the best discriminating power between the resistant and susceptible control crosses, and so among the tested progenies [46].

The QTL mapping study included 102 BSR pre-nursery screening test trials, spanning 10 years of data collection performed between 2007 and 2017. The trials encompassed the evaluation of 3910 GA × GB crosses, derived from 2069 and 361 individuals from the GA and the GB respectively. Each GB/GA parent included in the analysis was progeny tested on average 20/3.5 times respectively.

#### 2.1.4. Molecular Data

The mapping population was genotyped using a custom Affymetrix Axiom^®^ SNP array (Thermo Fisher Scientific, Waltham, MA, USA) containing 67,339 SNP markers. The CIRAD 67K SNP array was designed at CIRAD (Montpellier, France) and described in more detail in [44]. The genomic DNA of the 670 individuals (478 Deli and 192 La Mé) was extracted from freeze-dried oil palm leaf samples in the CIRAD DNA-bank using an automated method on a Biomeck FXP (Beckman Coulter, Brea, CA, USA) using the NucleoMag Plant Kit (Macherey-Nagel, Düren, Germany). The quantity of DNA extracted was measured with a Fluoroskan Ascent FL fluorometer (Thermo Fisher Scientific, Waltham, MA, USA); genomic DNA quality was checked with 1% (*w*/*v*) agarose gel electrophoresis and normalized at 100 ng/µL for further processing at the Gentyane genotyping platform. For each sample, 200 ng of genomic DNA were used; after whole genome amplification and fragmentation, 1500 µg were hybridized to arrays using the Affymetrix GeneTitan platform following Affymetrix guidelines available at http://www.affymetrix.com/, accessed on 2 June 2026. The Axiom Analysis Suite version 4.0 was used to process raw hybridization intensity data, clustering and genotype calling, following Affymetrix recommendations (https://downloads.thermofisher.com/Axiom_Analysis_Suite_v_4.0.1_User_Guide.pdf, accessed on 2 June 2026). SNP data were stored and managed in the oil palm database hosted in the Gigwa application (https://gigwa.southgreen.fr/gigwa/, accessed on 2 June 2026) [56].

#### 2.1.5. Methodological Framework

Traditional single-locus or multi-locus approaches, such as stepwise or backward selection, used for QTL mapping in complex populations or association panels, are often criticized for introducing biases in parameter estimates and inconsistencies in model selection [57,58]. To overcome these limitations, penalized likelihood approaches have been proposed to analyze all markers simultaneously within a single model [59]. By jointly accounting for all explanatory variables, these methods reduce spurious associations and enhance the detection of weak effects, making them particularly suited for situations with strong multicollinearity or where the number of predictors exceeds the number of observations. Notable examples include Ridge and Lasso regressions [60,61], as well as their Bayesian counterparts which utilize shrinkage priors on regression coefficients to incorporate biological knowledge and provide modeling flexibility [62]. In this study, we employed Bayesian variable selection (BVS) implemented in the R [63] package BGLR [49] version 1.1.4. While BGLR offers various models with different prior distributions to shrink small effects toward zero (e.g., Bayesian Ridge Regression with a Gaussian prior, BayesA with a scaled-t prior, BayesB, or Bayesian Lasso with a double-exponential prior), we specifically used the BayesC model. This approach relies on a “spike-and-slab” prior, which is a two-component mixture composed of a point mass at zero (the spike) and a Gaussian distribution (the slab), allowing large effects to remain substantial while effectively filtering out negligible ones.

- Statistical models

We followed a two-step procedure for QTL detection. The first step was dedicated to estimating non-genetic effects on the complete dataset (*n* = 3910 crosses). The second step was the actual QTL detection, performed on a subset of the dataset consisting of crosses for which both parents GA and GB were genotyped (*n* = 1092).

For the first step, assuming *Y*, the percentage of affected individuals per cross at the end of the trial, to be normally distributed, we used the following linear mixed model:

(1) $$Y=\mu +X_{C}\beta_{C}+X_{T}\beta_{T}+Z_{A}u_{A}+Z_{B}u_{B}+e,$$

where $X_{C}$ is a n×m design matrix relating observations to *G. boninense* clonal line fixed effects $\beta_{C}$ where $\beta_{C}$ is a m×1 vector (m=7); $X_{T}$ is a n×m design matrix relating observations to trials fixed effects $\beta_{T}$ where $\beta_{T}$ is a m×1 vector (m=102); and $Z_{A}$ and $Z_{B}$ are $n\times q_{A}$ and $n\times q_{B}$ design matrices relating observations to general combining ability (GCA) random effects for GA and GB, $u_{A}\sim N\left(0,A_{A}\sigma_{A}^{2}\right)$ and $u_{B}\sim N\left(0,A_{B}\sigma_{B}^{2}\right)$ respectively, where $u_{A}$ and $u_{B}$ are $q_{A}\times 1$ and $q_{b}\times 1$ vectors respectively ($q_{A}$ = 2069 and $q_{b}$ = 361). $A_{A}$ and $A_{B}$ are the pedigree-based kinship matrices of GA and GB respectively. Finally, e is an n×1 vector of residuals assumed to follow a Gaussian distribution $N\left(0,\sigma^{2}I_{n}\right)$ independent of $u_{A}$ and $u_{B}$.

For the second step, the following QTL model was used with the non-genetic effects estimated in step 1 treated as fixed effects:

(2) $$Y=\mu +X_{C}\beta_{C}+X_{T}\beta_{T}+X_{A}\beta_{A}+X_{B}\beta_{B}+e,$$

where $X_{A}$ is the n×m SNP matrix (m=21,481) and $\beta_{A}$ the effects associated to SNP markers for the GA parents; $X_{B}$ is the n×m SNP matrix (m=30,913) and $\beta_{B}$ the effects associated to SNP markers for the GB parents; and e the n×1 vector of residuals assumed to follow a Gaussian distribution $N\left(0,\sigma^{2}I_{n}\right)$. For each GA and GB, SNPs included in a reference genetic maps built in a previous study [44] were used in the analysis after a MAF filtering at 5% in each genetic group.

In order to select the most relevant positions, we used the BayesC prior, a shrinkage prior on markers effects associated to both GA and GB. The probability of a marker having a non-zero effect (probIn) was set to 0.0001; the scale parameter of the regularizing variance component was configured with a scale shape of 0.55 and a rate of 10^−4^ (counts = 2). Parameter estimates were obtained by running out three times the Markov chain Monte Carlo (MCMC) algorithm with 300,000 iterations, the first 30,000 being used as burn-in, and a thinning of 15. From each run, we obtained posterior inclusion probabilities (PIPs) for each marker that enabled the variable selection and subsequently the identification of QTL regions.

- Identification of QTL intervals and variance component analysis

Following the inference of the QTL model, the PIP values were obtained for three independent MCMC runs and averaged by marker and by genetic group GA and GB. The average PIP values were then summed over a 3 Mb sliding window to identify QTL intervals while mitigating variations in SNP density across the genome. A threshold of 0.2 * max (summed PIP value) was applied to declare a QTL. Because the distribution of PIPs under the null hypothesis is unknown, permutation tests were performed to empirically validate the threshold. For each of the 1000 replicates, the phenotypic trait (Y, representing the percentage of affected individuals per cross at the end of the trial) was randomly shuffled. Each permuted dataset was then analyzed using the BGLR package, maintaining the same parameterization as the original QTL mapping analysis. The maximum PIP value obtained in each replicate was recorded to construct the empirical null distributions, both for individual SNPs and for summed PIPs within 3 Mb sliding windows (Figure S2). Finally, the 95%, 99%, and 99.9% percentiles were calculated from these null distributions and indicated on the QTL plots.

To define QTL confidence intervals, neighboring SNPs were added sequentially in order of physical distance from the top SNP until their cumulative PIP reached 95% of the peak 3 Mb window aggregated PIP used for QTL detection. The flanking SNPs defined the borders of each confidence interval (Table S3).

To estimate the variance explained by each identified QTL, individual genomic breeding values were calculated from the posterior mean marker effects estimated by BGLR. The genomic variance associated with each marker set was defined as the variance of these predicted genomic values. For genomic models partitioned into two parental effects with their respective SNP matrices, the total genomic variance was calculated from the combined genomic predictions using two Bayesian ridge regression (BRR) priors in BGLR, thereby accounting for the covariance between the two genomic components. The residual variance $V_{e}$ was obtained directly from the posterior estimates provided by BGLR. The proportion of phenotypic variance explained by each genomic component PVE was calculated as:

$$PVE=\frac{V_{g}}{V_{g}+V_{e}}$$

where $V_{g}$ represents the variance associated with the genomic component of interest. The same procedure was applied to two different models: (i) a genomic model including all available SNP markers within the Group A (GA) and Group B (GB) genomic datasets, and (ii) a QTL model including only SNP markers located within the identified QTL intervals. Comparing these two models allowed us to estimate the proportion of the total phenotypic variance (PVE) and the proportion of total genomic variance (GVE) captured by the QTL-associated markers (Table S3).

- QTL haplotype diversity and effects

To identify the haplotypes segregating at each QTL and track them back to the founder’s haplotypes, a semi-manual haplotype reconstruction was performed. For this purpose, we used all the genotyping data obtained using CIRAD 67k SNP array on more than 8000 individuals from the breeding program, including the QTL mapping and RNA-seq populations from this study. Genotypic data were phased using Beagle v5.5 [64]. For each QTL, haplotypes were identified from SNP markers within a 1-to-3 Mb window centered on the top SNP. The 30 most frequent haplotypes, initially labeled H1–H30, were compared to the haplotypes of founder individuals. Those sharing at least 90% similarity were clustered and renamed according to their genetic origin (DE for Deli and LM for La Mé), with the smallest numbers corresponding to the highest frequency in the breeding population (for example, DE1 refers to the most frequent haplotype in the Deli population). In this study, particular attention was given to the founders of the GA group D2, D6, and D7 and the founder of the GB group LM1, as these founders provided the genetic background of the RNA-seq population.

For the validation and characterization of haplotypes and their effects, parental GCA were predicted using an updated dataset (*n* = 7672 crosses) with additional data collected from 2017 to 2024 and an animal model which allows the integration of GA × GA and GB × GB crosses. Assuming y, the number of affected individuals per plot at the end of the trial, to follow a binomial distribution of the parameter p, we used the following generalized linear mixed model with a logit transformation η=log(p/(1−p)) in ASReml-R v4.2 [65]

(3) $$\eta =\mu +X_{c}\beta_{C}+Z_{T}T+Z_{R}R+Z_{C}C+Z_{A}u_{A}$$

where η is a n×1 vector of observation at logit scale (n=88,701); $X_{c}$ is a n×m design matrix relating observations to *G. boninense* clonal line fixed effects $\beta_{C}$ where $\beta_{C}$ is a m×1 vector (m=8); $Z_{T}$, $Z_{R}$ and $Z_{C}$ are the n×s, n×v and n×w design matrices associated with the trial, the replicate within the trial and the cross effects, $T\sim N(0,\sigma_{T}^{2}I)$ with T being a s×1 vector (s=191), $R\sim N(0,\sigma_{R}^{2}\;I)$ with R being a v×1 vector (v=955), and $C\sim N(0,\sigma_{C}^{2}\;I)$ with C being a w×1 vector (w=7672); and $Z_{A}$ is a $n\times q_{A}$ design matrix relating observations to the genetic additive random effects for tested progenies, $u_{A}\sim N(0,A_{A}\sigma_{A}^{2})$, where $u_{A}$ is a $q_{A}\times 1$ vector ($q_{A}=7672$). $A_{A}$ is the pedigree-based kinship matrix of the tested progenies.

### 2.2. RNA Sequencing (RNA-Seq) Approach

#### 2.2.1. Plant Material

In 2019 at Tanah Gambus PT Socfindo estate, a dedicated trial was conducted in parallel with the ongoing pre-nursery trials, involving eight Deli × La Mé crosses selected based on their BSR resistance level (susceptible, intermediate, or resistant) and their genetic relatedness to the QTL mapping population. Six seedlings were grown for each cross selected, three of them planted in a polybag with an inoculated RWB (I) as the classical inoculation scheme of pre-nursery crosses [47], and the three others with a non-inoculated RWB (NI) as control (Figure S3). Seedlings were grown for three weeks until the roots were in contact with the RWB (Figure S3). At that time, the 48 seedlings from the selected crosses were carefully removed from the soil, the roots were successively washed in water, in 70° alcohol and in water again (Figure S3). The roots and the bole were harvested for each individual sample and cut into fine pieces transferred into a 1.5 mL microtube containing 1 mL of a conservation solution of RNA later^®^ (Qiagen, Germantown, MD, USA or Ambion, Austin, TX, USA). The samples were stored at −20 °C until RNA extraction. Leaves sampled for genotyping were stored in silica gel prior to DNA extraction. The 48 seedlings were genotyped using the same 67K SNP array used for the QTL mapping population. Haplotypes at GA and GB were identified for each seedling following the method described in Section 2.1.5. The identification of haplotypes enabled the legitimacy of the sequenced seedlings to be checked, leading to the removal of 18 suspicious seedlings from the study, thereby leaving 30 seedlings for the subsequent analyses.

#### 2.2.2. RNA Extraction, Construction of the cDNA Libraries and RNA-Seq

For each of the 48 seedlings sampled, approximately 100 mg of root tissue was ground in a mortar with liquid nitrogen, while bole tissue was ground using the Geno Grinder 2010 system (SPEX SamplePrep, Metuchen, NJ, USA). The Geno Grinder parameter used was 1500 rpm for 1 min, repeated two or five times for oil palm tissues. For the bole powder, 350 µL of TE3D extraction buffer (14.8 g EDTA, 84.4 g Tris, 20 g Nonidet P-40, 30 g lithium dodecyl sulfate, 20 g sodium deoxycholate, 95 mL H_2_O) was added, while 350 µL of MR1 extraction buffer from the NucleoMag Plant RNA isolation kit (Macherey-Nagel, Düren, Germany) was used for the roots. After 20 min of incubation at room temperature, RNA extraction protocol was followed according to the NucleoMag Plant Kit manufacturer’s instructions for use with the KingFisher system (Thermo Fisher, Waltham, MA, USA; https://www.thermofisher.com). RNA integrity number (RIN) and quantification of total RNA were measured using the Agilent 4200 TapeStation System (Agilent Technologies, Santa Clara, CA, USA). A total of 96 RNA-seq libraries were prepared according to the Illumina protocol with the TruSeq Stranded mRNA Library Prep Kit (Illumina, San Diego, CA, USA), using 1 µg of total RNA. The indexed libraries were pooled in 24-plex and subjected to pair-end 2 × 150 bp sequencing on an Illumina HiSeq4000 (Genewiz, South Plainfield, NJ, USA). Each pool of 24 libraries was run in parallel on four lanes, allowing a technical replicate. RNA sequences were submitted to the EBI ENA database and are available at study PRJEB113782.

#### 2.2.3. Genomic Analyses

A new African oil palm reference genome was used in this study (Lopez et al., in prep). In brief, one genotype from the La Mé population was sequenced using a combination of PacBio Hifi reads and Hi-C sequencing. The assembly was carried out using hifiasm v0.16.1 [66,67,68]. The assembled sequences were then anchored in 16 linkage groups using Lep-Anchor v2024.05.21 [69]. This final assembly was then annotated using the Eukaryotic Genome Annotation Pipeline—External (EGAPx, https://github.com/ncbi/egapx, accessed on 2 June 2026) v0.4.1 with a combination of RNA-seq data produced in this study along with unpublished La Mé isoseq transcriptomic data (Lopez et al. in prep). The pipeline predicted 22,485 proteins that were functionally annotated using an in-house workflow. The predicted proteins were searched for similarities with known transposable elements using blast, Hmm profiles and associated GO terms. Functional annotation was obtained by running InterProScan v5.67-99 [70,71], KofamScan v1.3.0 [72] and EggNog v5.0.2 [73] database searches. Genomic sequence and gene annotation were submitted to the EBI ENA database and are available at accession ERZ29504525 under study PRJEB113782.

RNA-seq reads pairs were submitted to QC analysis using FastQC 0.11.7 [74]. The 48,150 bp paired-ends libraries accounted for 818 million reads that ranged from 12 million up to 31 million reads per pair. Average mean quality was 38.05 (min. 37.3; max. 38.4). The percentage of bases >Q30 ranged from 88.7% to 92.7% with an average of 91.3%. Cutadapt (v4.9) [75] was used to find and remove Illumina Trueseq adapter sequences, remove low quality base-pair in both 5′ and 3′ of each read based on a quality cutoff of 20, and discard resulting pairs of reads if one of the reads was shorter than 35 bases. The nextseq-trim option was also used to handle poly-g tails, using a cutoff of 20. Reads were mapped using Hisat2 (v2.2.1) [76] onto the La Mé reference genome using the following parameters: “--sensitive”, “--rna-strandness RF” and “--max-intronlen 25,000. Read counting was performed using FeatureCount from the subread package (v2.0.6) [77] using the “-M --fraction” parameter to handle multi-mapping reads.

#### 2.2.4. Differentially Expressed Gene (DEG) Analysis

RNA-seq gene counts were analyzed in R version 4.4.1 using the Bioconductor package DESeq2 version 1.46.0 [78]. Genes with a total count of 10 or less across all samples were excluded from further analyses. Library-size normalization was performed using the median-of-ratios method implemented in DESeq2. Since RNA-seq count data follow a non-normal negative binomial distribution, and to satisfy the Gaussian assumption of downstream Bayesian regression models, normalized counts were log2-transformed (log2(normalized count+1)). These transformed expression levels were subsequently used as a continuous response variable (Y) for Bayesian variable selection using the BGLR R package version 1.1.4 [48]. Our experimental design for the RNA-seq study included multiple sources of variation: treatment (inoculation with *G. boninense* or not), organ (root or bole), haplotypes at segregating QTLs and their interactions. QTL effects were included in the analysis when there were at least 10 occurrences of each haplotype among the sequenced individuals, which excluded the QTLs GA_06at88 and GB_08at23 from the analysis. Considering all the predictors included in the analysis, the normalized and transformed counts were independently analyzed for each of the 22,485 genes according to the following model:

(4) $$Y_{g}=\mu +X\beta_{g}+e,$$

where $Y_{g}$ is the n×1 vector of normalized and transformed count of the gene g (n=60), X is the n×m predictor matrix (m=55), $\beta_{g}$ is the predictor effects for the gene g, and e is the n×1 vector of residuals following a Gaussian distribution $N\left(0,\sigma^{2}I_{n}\right)$. The m predictors correspond to treatment (T), organ (O) and haplotypes at QTL (Q) and the interactions T*O, T*Q, O*Q et T*O*Q.

Following the approach used for QTL detection, we applied a Bayesian variable selection model using the BayesC prior on predictor effects in order to identify the best model associated with each gene. The probability of a predictor having a non-zero effect (probIn) was set to 0.01; the scale parameter of the regularizing variance component was configured with a scale shape of 0.55 and a rate of 10^−4^ (counts = 2). Parameter estimates were obtained by running out two times the Markov chain Monte Carlo (MCMC) algorithm with 12,000 iterations, the first 3000 being used as burn-in. From each run, we obtained posterior inclusion probabilities (PIPs) for each predictor that enabled the variable selection considering a PIP threshold of 0.6. Above this threshold, a gene was considered as a DEG relatively to the predictor considered.

#### 2.2.5. Candidate Gene Prioritization Within the QTL Intervals

For each gene on a chromosome containing a QTL, the distance from the top SNP of the closest QTL was calculated based on physical positions. Differentially expressed genes (DEGs) located within or outside a 3 Mb window centered on this top SNP were classified as cis-DEGs or trans-DEGs respectively. The DEGs associated with predictors involving QTL effects (Q, T*Q, O*Q, and/or T*O*Q, see Section 2.2.4) were subjected to further analyses to shed light on the mechanisms and candidate genes involved in BSR resistance. Gene Ontology (GO) terms enrichment analysis was performed on the DEG lists using clusterProfiler R package version 4.14.6 [79]. Gene co-expression networks were built based on cis-DEGs and trans-DEGs related to the QTL effects (predictors Q, T*Q, O*Q and/or T*O*Q) using WGCNA R package version 1.74 [80]. Finally, for cis-DEGs, the effects of QTL haplotypes on their expression profiles were examined and compared to the effects of QTL haplotypes on BLUPs for BSR resistance (see Section 2.1.5). An R Shiny application has been developed to visualize the effects of QTL haplotypes for all the genes analyzed; it is publicly available at https://shinyapps.southgreen.fr/app/rnaseq-qtl-daval-et-al, accessed on 2 June 2026. All information used for the candidate gene prioritization are available in Table S3.

## 3. Results

### 3.1. Segregation of BSR Resistance in the Breeding Population

**Figure 1:**
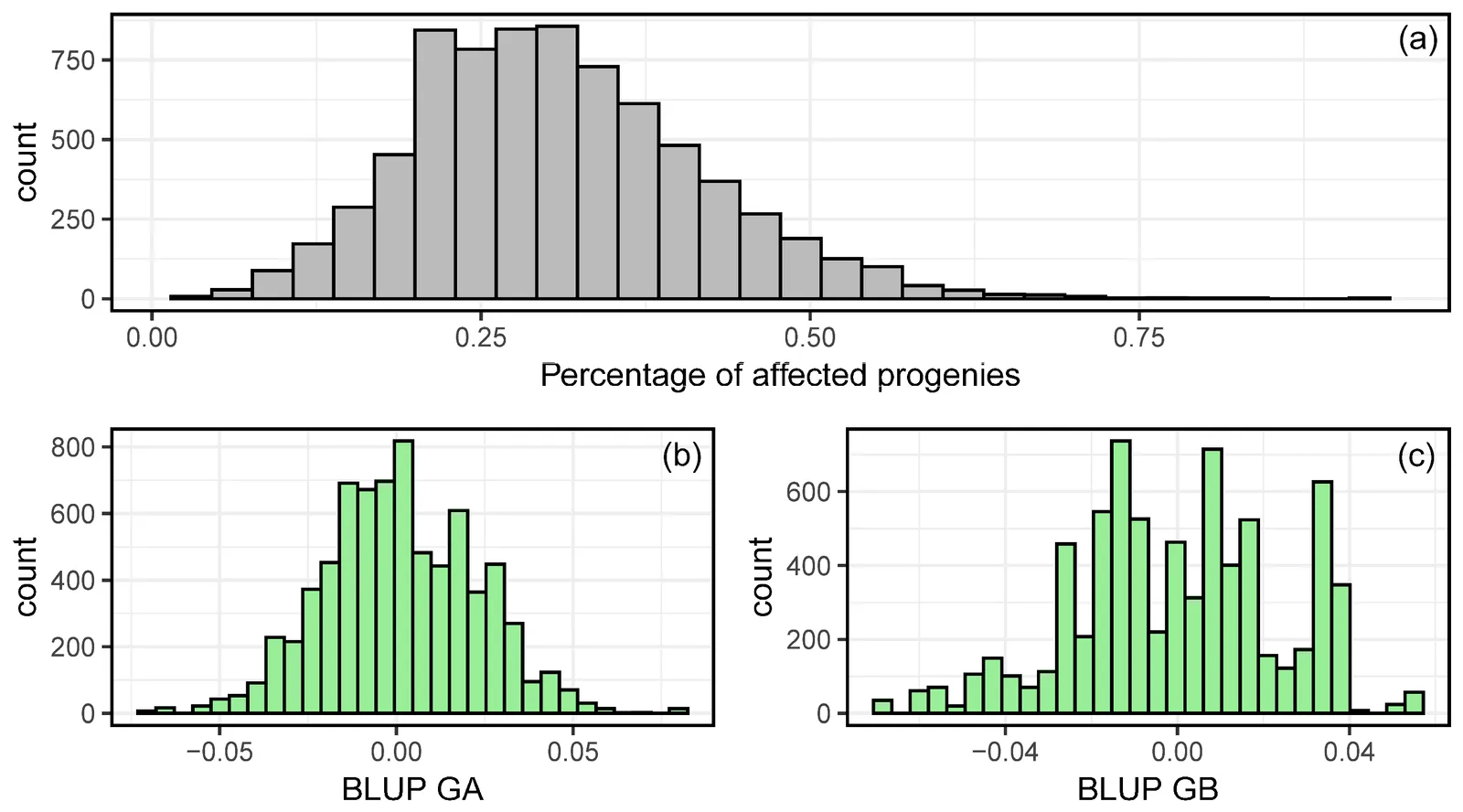
Distribution of resistance to *Ganoderma boninense* in the population tested in pre-nursery trials: (**a**) Raw distribution of the percentage of affected progenies per cross; (**b**) Distribution of BLUPs values for GA parents directly involved in pre-nursery trial; (**c**) Distribution of BLUPs values for GB parents directly involved in pre-nursery trial.

The resistance to *G. boninense* was evaluated in pre-nursery trials involving 3910 GA × GB oil palm crosses derived from 2069 GA and 361 GB parents, based on an incomplete mating design. At the end of the trial, on average 30.8% of oil palm seedlings per cross presented disease symptoms, with incidence ranging from 3% to 92.5% among crosses (Figure 1a). The BLUP values (see Section 2.1.4, Equation (1)) revealed additive genetic variation for the proportion of progenies affected by *G. boninense* for each parental group. BLUPs of parental effects ranged from –0.07 to 0.07 for GA (Figure 1b) and from −0.068 to 0.055 for GB (Figure 1c). Parents with negative BLUP values were associated with a lower proportion of affected progenies, indicating the presence of favorable genetic effects contributing to resistance, whereas positive values were associated with increased susceptibility.

### 3.2. Genetic Bases of BSR Resistance in Pre-Nursery Trials

**Figure 2:**
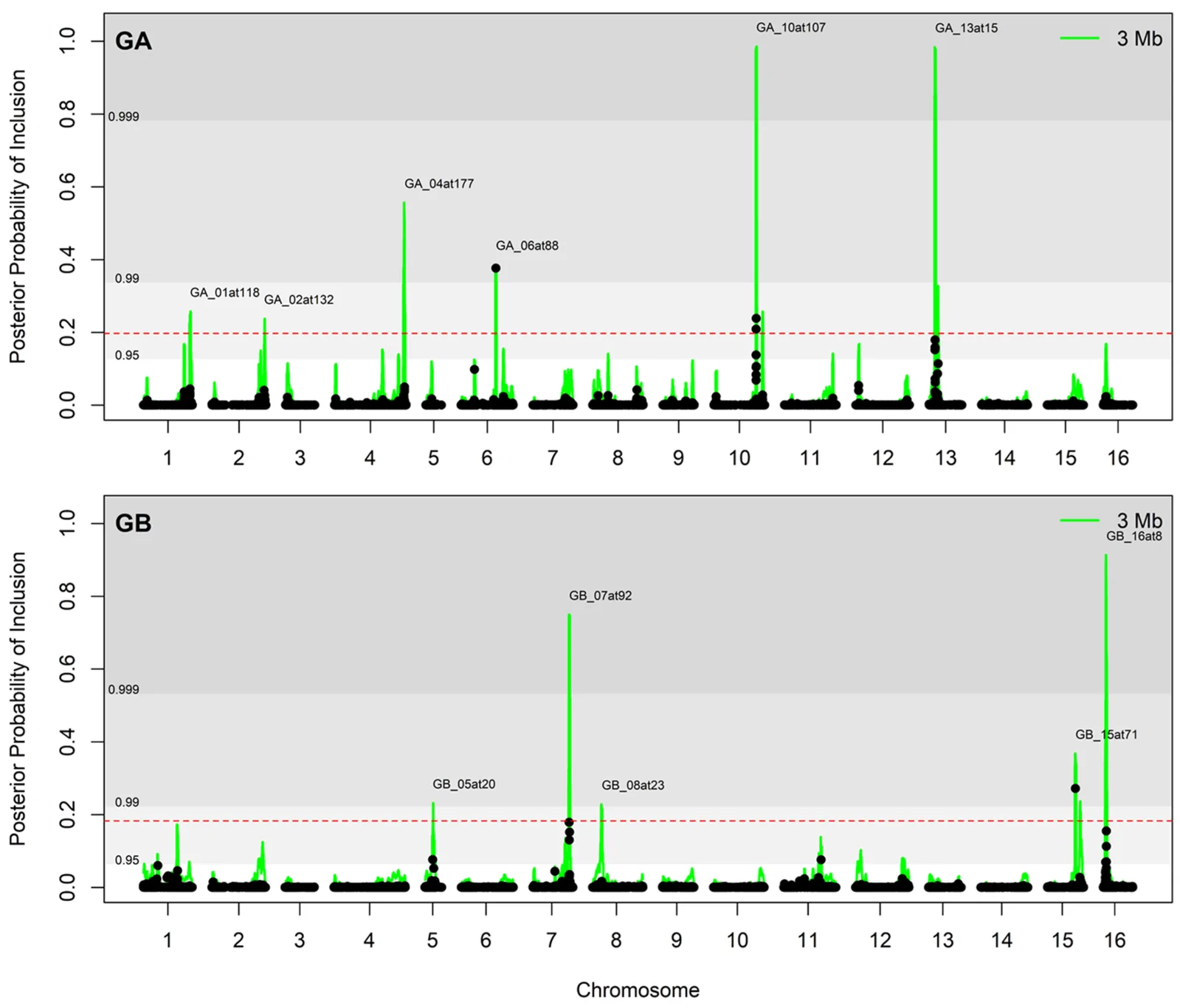
QTL mapping of basal stem rot (BSR) resistance in the Deli (GA, top panel) and La Mé (GB, bottom panel) oil palm populations. Black circles represent individual SNP posterior inclusion probabilities (PIPs) plotted across the genome. Green lines show aggregated PIPs calculated within 3 Mb sliding windows, with the associated significance threshold indicated by a red dotted line (see Section 2.1.5). Grey shading represents the 95%, 99%, and 99.9% quantiles of the empirical null distribution for the 3 Mb window aggregated PIPs. Identified QTL names are positioned above the top SNP (highest-PIP) for each detected locus.

QTL mapping of BSR resistance in the GA and GB populations was performed using a Bayesian variable selection (BVS) approach. A total of 11 QTLs were identified: 6 and 5 in the GA and GB populations respectively (Figure 2, Table S2). In the GA population, QTLs were identified on chromosomes 1, 2, 4, 6, 10, and 13. The strongest signals were located on chromosomes 10 and 13, cumulative PIPs close to 1, whereas the highest PIP for an individual SNP was detected on chromosome 4. In the GB population, QTLs were detected on chromosomes 6, 7, 8, 15 and 16, with the strongest signal observed on chromosome 16 (Figure 2). QTLs did not colocalize between the two heterotic groups, suggesting that distinct genomic regions contribute to BSR resistance in the GA and GB genetic backgrounds. Moreover, the contribution of the QTL differed markedly between the two genetic groups: the GA QTL had a genomic variance more than three times superior to GB QTL, capturing a much higher percentage of the total genomic variation estimated genomic prediction model (Table S2). Among the GA QTL, the loci GA_13at15 and GA_10at107 showed the largest individual contributions, whereas GB_16at8 was the stronger QTL for GB (Table S2).

**Figure 3:**
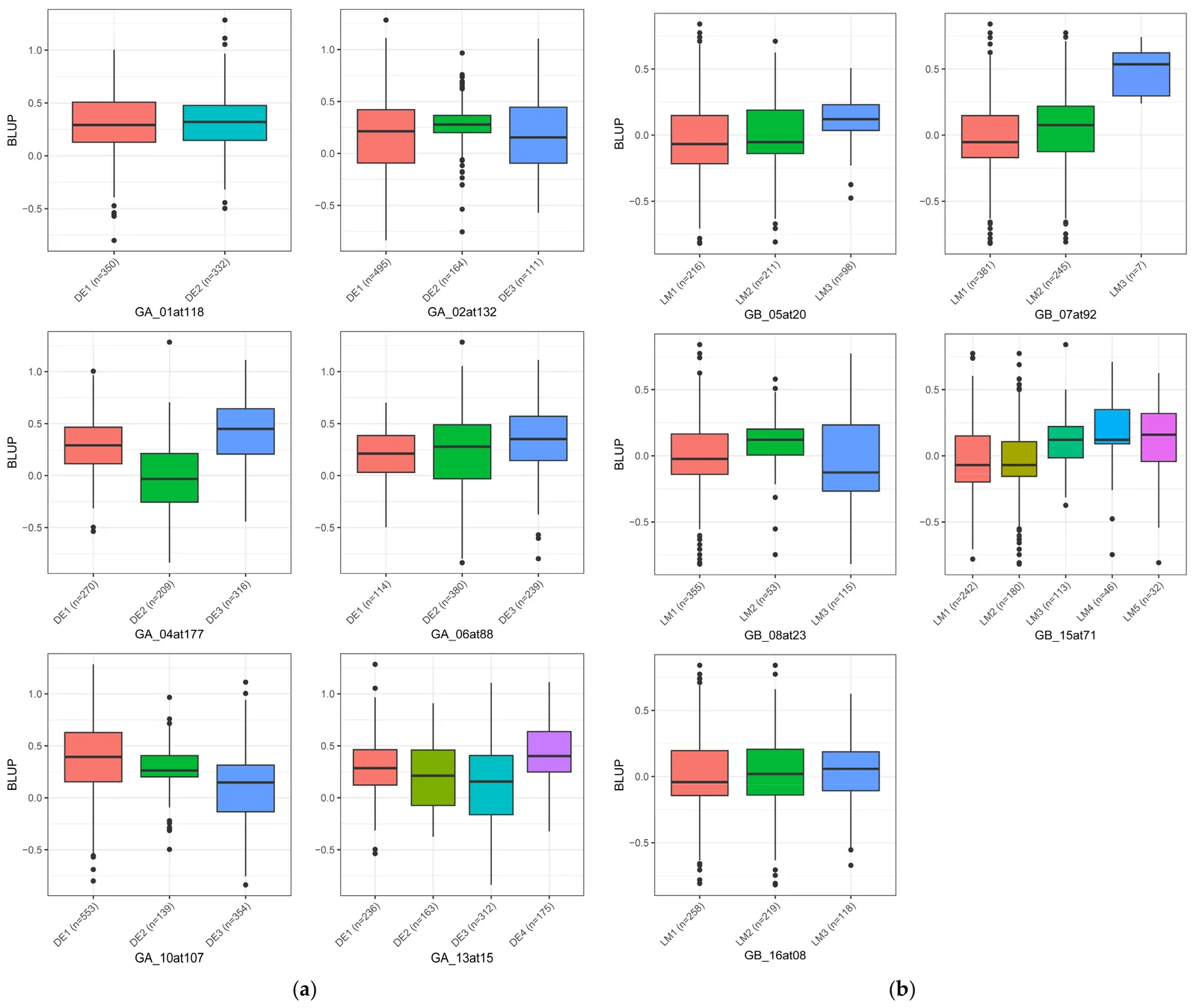
Effects of QTL haplotypes on BSR resistance: boxplots representing BLUPs (see Section 2.1.5) for each GA (**a**) and GB (**b**) QTL by haplotype identified. The number of haplotypes in the parental population is given in parenthesis.

Haplotype characterization was performed within the parental populations for each QTL, enabling the determination of their genetic origins. Haplotype effects were then visualized and validated using parental GCA values (BLUPs) predicted from the updated dataset (see Section 2.1.4). Most of the QTLs in the Deli population had three haplotypes—4 out of 6—with the exception of QTL GA_01at118, which had two haplotypes, and QTL GA_13at15, which had four haplotypes. This was similar for the QTL of La Mé population, with 4 out of 5 QTLs exhibiting three haplotypes, except for GB_15at71 with five haplotypes (Figure 3b). In the Deli population, clear segregation of haplotype effects was observed at QTLs GA_04at177, GA_06at88, GA_10at177 and GA_13at15 (Figure 3a). Resistant haplotypes were identified as DE2 for GA_04at177 and DE3 for GA_10at107, while susceptible haplotypes were DE3 for QTL GA_04at177 and GA_06at88 and DE4 for GA_13at15. In the La Mé population, clear segregation of haplotype effects was observed at QTLs GB_05at20, GB_07at92, GB_08at23 and GB_15at71, whereas no resistant haplotype could be determined for QTL GB_16at08 (Figure 3b). The susceptible haplotypes were the most clearly defined in the distributions, namely LM3 for GB_05at20 and GB_07at92 QTL and LM3, LM4, and LM5 for GB_15at71.

### 3.3. Transcriptomic Analysis of the Response to G. boninense Inoculation

**Figure 4:**
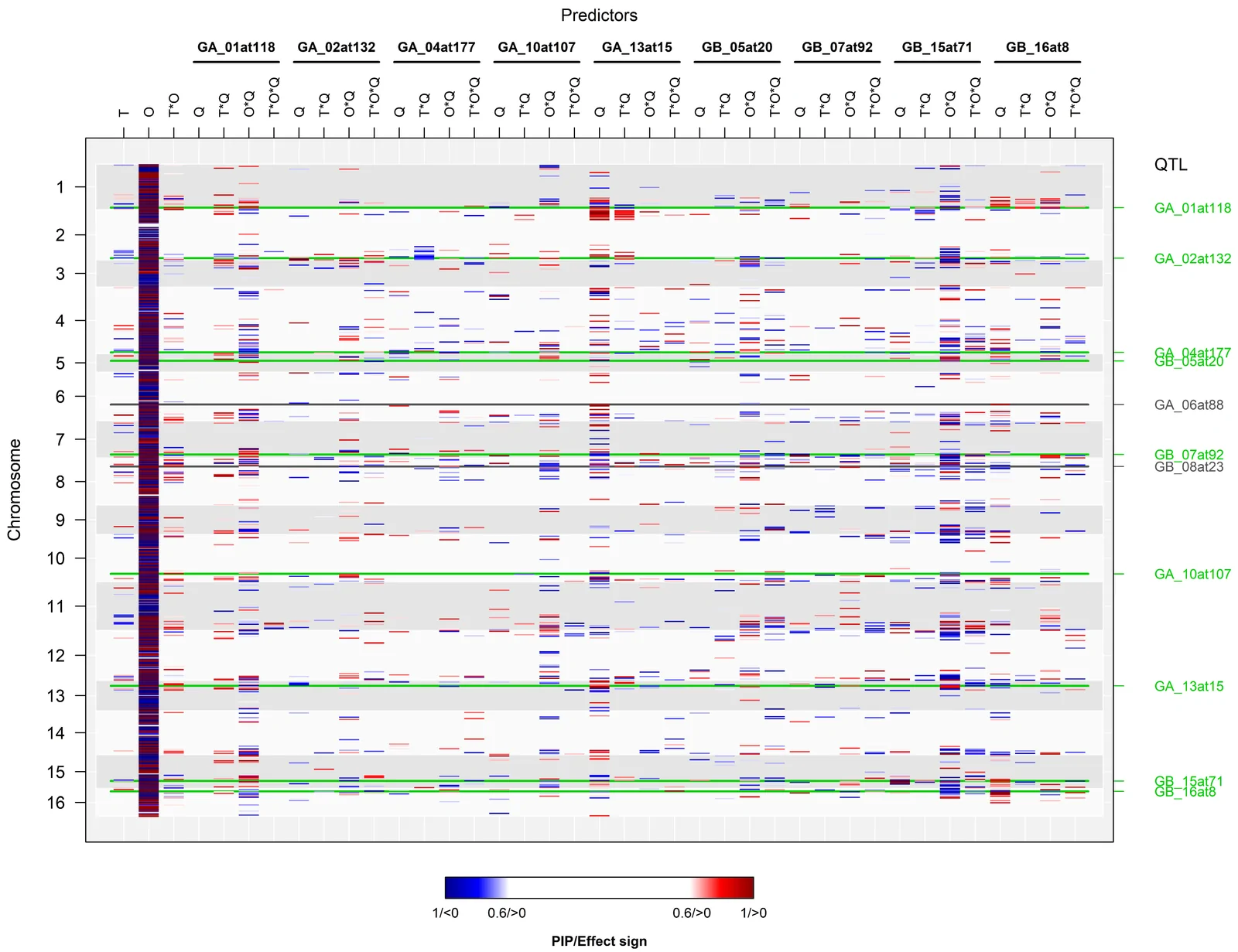
Heatmap of differentially expressed genes (DEGs) in the RNA-seq population. The heatmap visualizes the results of a Bayesian variable selection analysis, considering the 16 chromosomes (y-axis) and a matrix of genetic and environmental predictors (x-axis). Each gene is represented by a horizontal segment at its physical position in the La Mé genome. The color scale associated with the genes represents the posterior probability of inclusion (PIP) for the given predictor, plotted on the x-axis, with a threshold of 0.6 for gene display. Stronger color saturation indicates a higher PIP (e.g., strong red/blue), while paler shades (visible down to a PIP of 0.6, where the color scale changes) indicate lower PIPs. Blue and red indicate the directions of effects. QTLs regions are represented by horizontal lines at their physical positions, in green for QTLs that segregate in the RNA-seq population and in gray if they do not segregate. T: treatment effect; O: organ effect; Q: QTL effect.

Of the 22,485 genes surveyed via RNA-seq, 11,940 were identified as differentially expressed (DEGs) using a Bayesian variable selection approach. These DEGs respond to predictors based on the experimental factors: non-genetic, treatment (T) and organ (O), and genetic, QTL haplotype (Q), and their interactions (Figure 4). The distribution of DEGs was uniform across gene-rich regions of the genome, with no clear presence of DEG hotspots (Figure 4). However, when DEGs associated with organ-specific effects are excluded, the QTL regions GA_02at32, GA_04at177, GB_07at92, and GB_15at71 show a slight enrichment (Figure 4 and Figure S4).

**Figure 5:**
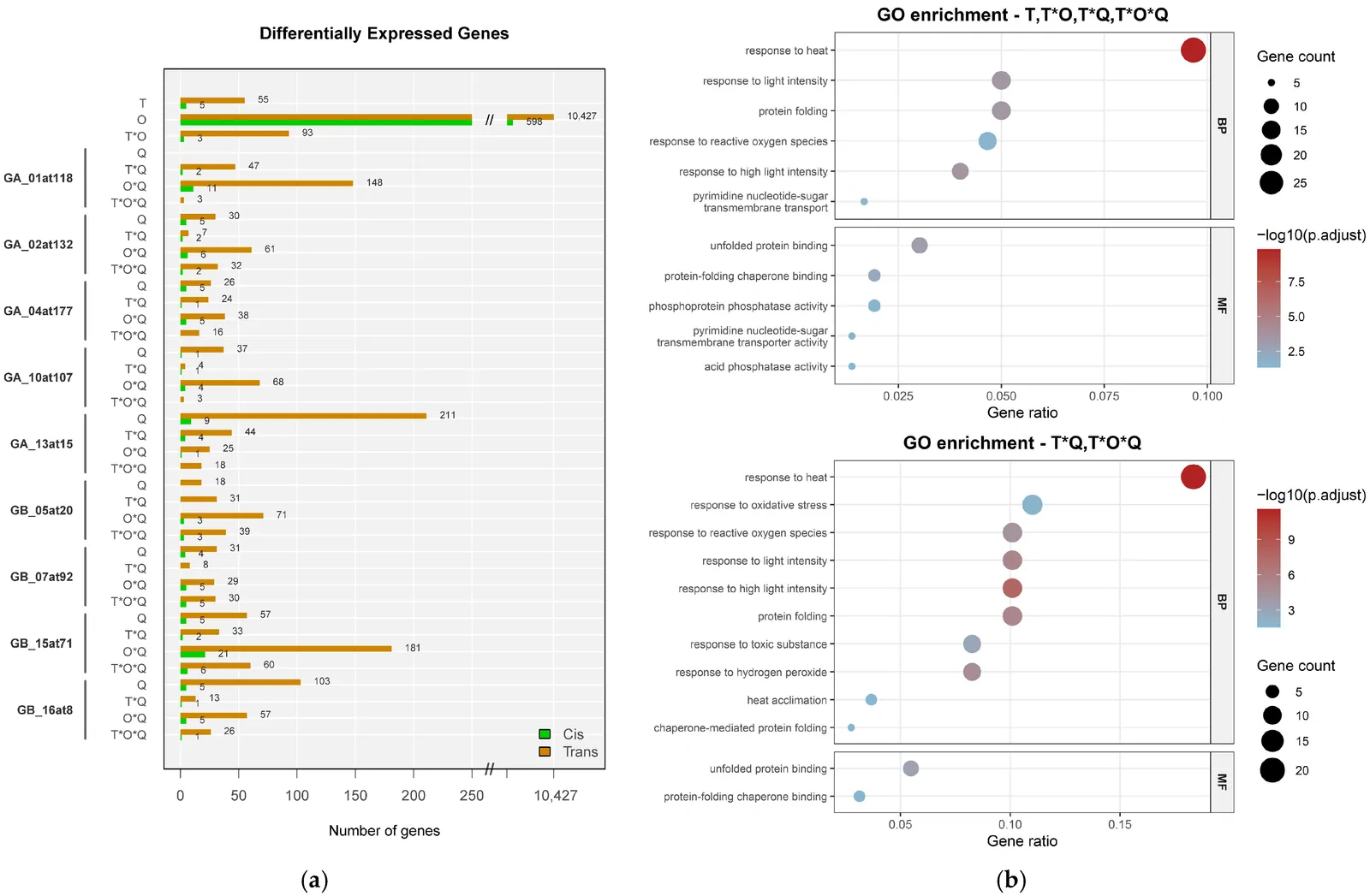
Characterization of differentially expressed genes (DEGs) obtained with the Bayesian variable selection (BVS): (**a**) Number of cis-DEGs (green) or trans-DEGs (orange) relative to the predictors used in the BVS model (“T”: treatment, “O”: organ, “Q”: QTL and their interactions); (**b**) Gene Ontology (GO) term enrichment for the DEGs relative to treatment and the interaction with treatment (top panel) and DEGs relative to treatment × QTL interactions (bottom panel). BP: biological process; MF: molecular function.

Most of the DEGs—11,025 in total—exhibited constitutive differential expression between the roots and the bole (Figure 5a). A total of 551 DEGs were identified in association with all predictors related to treatment effect (T, T*O, T*Q, and T*O*Q), including 195 involved in interactions with QTL haplotypes (Figure 5a). Enrichment analysis of Gene Ontology (GO) terms for DEGs involved in the treatment response revealed an overrepresentation of genes involved in responses to stresses: heat, reactive oxygen species (ROS), or high light intensity (Figure 5b). By focusing on the DEGs involved in treatment × QTL interactions, the same categories were found, along with the response to toxic substances (Figure 5b).

Considering QTL intervals of 3 Mb centered on the top SNP, the DEGs were classified as cis-DEGs or trans-DEGs depending on whether they were within or outside the interval respectively (Figure 5a). Cis- and trans-DEGs were identified for all the QTL segregating in the RNA-seq population, with a few dozen of trans-DEGs per QTL (Figure 5a). On the other hand, a maximum of 5 cis-DEGs was identified per QTL among the 86 genes on average contained within each QTL interval, enabling the effective prioritization of candidate genes (CG) underlying the QTLs (Figure 5a).

### 3.4. Prioritization of Candidate Genes for BSR Resistance in QTL Intervals

**Figure 6:**
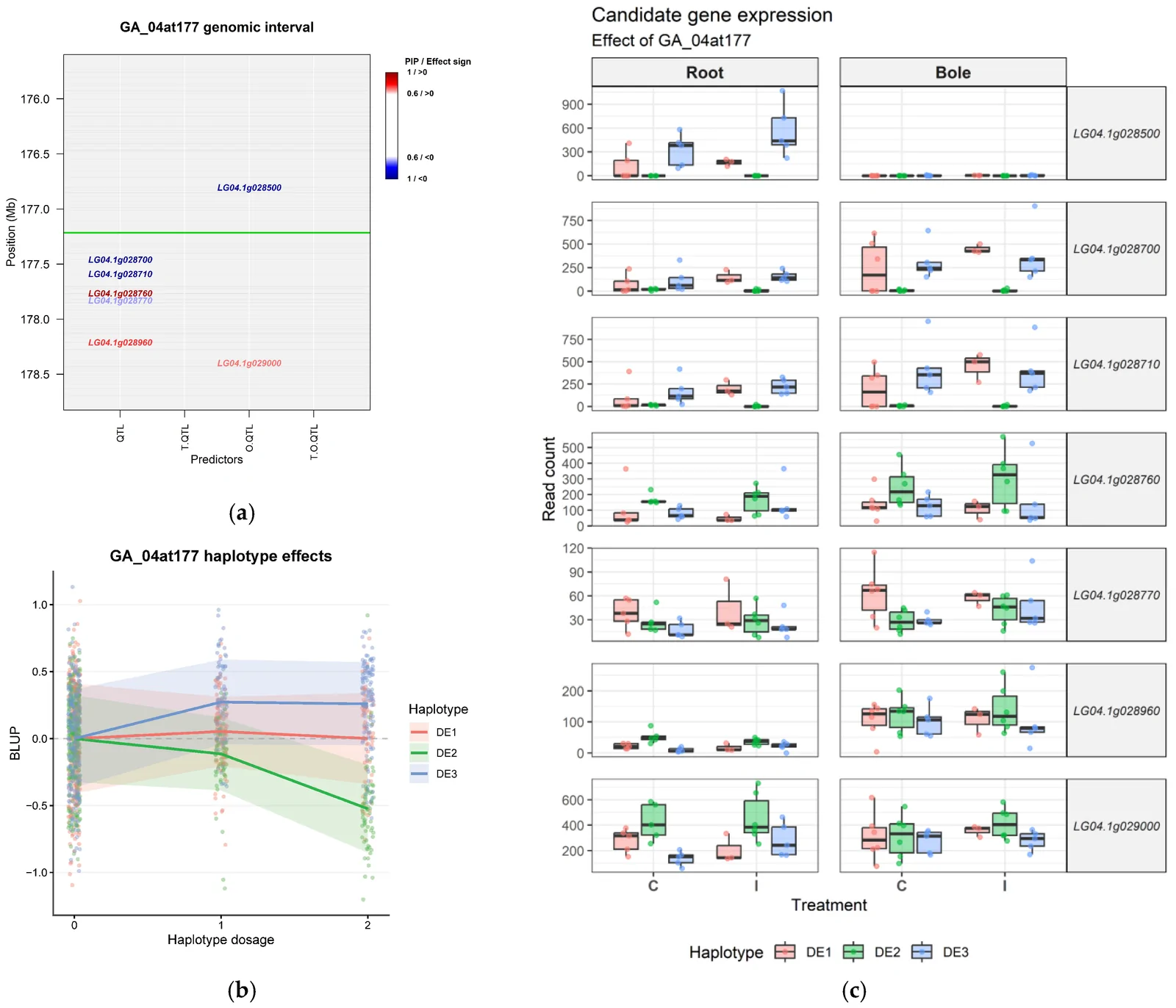
Prioritization of candidate genes in the QTL GA_04at177 genomic interval. (**a**) Heatmap of differentially expressed genes (DEGs) in the 3 Mb QTL interval centered on the top SNP (green line). The color scale for gene names represents the posterior probability of inclusion for the given predictor, plotted on the x-axis, with a threshold of 0.6 for gene display; blue and red indicate negative and positive effects respectively. Other genes in the interval are represented by horizontal grey lines at their physical positions. T: Treatment effect; O: organ effect; Q: QTL effect. (**b**) Effect of haplotype dosage at GA_04at177 QTL on BLUP for BSR resistance (*n* = 427, see Section 2.1.5). Each point represents a unique GA parent, and BLUP values are displayed normalized relative to the dosage = 0 for each haplotype. (**c**) Effects of GA_04at177 QTL haplotypes on the expression levels of the cis-DEG in the QTL genomic interval. C: Control, I: Inoculated.

To illustrate the ability of our approach—which combines QTL and RNA-seq—to prioritize candidate genes for BSR resistance, we focused on QTL GA_04at177 from Deli genetic background, for which two of the three haplotypes segregating in the RNA-seq population had strong opposite effects on resistance (Figure 6b). Among the 94 genes present within the 3 Mb QTL interval, 7 cis-DEGs were identified at distances ranging from 0.24 to 1.18 Mb from the top SNP (Figure 6a). The three genes closest to the top SNP also had the highest PIPs (>0.94) for QTL effects (LG04.1g028700 and LG04.1g028710) or organ × QTL effects (LG04.1g028500), with all three showing effects in the same direction (Figure 6a). Remarkably, gene LG04.1g028710, located at only 370 kb from the top SNP, had the highest PIP (0.42) for the treatment × QTL predictor among the 7 cis-DEGs (Table S3). Comparing the resistance levels of the three haplotypes segregating at QTL GA_04at177 (Figure 6b) with the expression patterns of the 7 cis-DEGs according to organ, treatment, and QTL haplotypes (Figure 6c), a strong relationship was observed for the same 3 genes, LG04.1g028500, LG04.1g028700, and LG04.1g028710: the highly resistant haplotype DE2 was not expressed in any tissue or under any treatment, whether inoculated with *G. boninense* or not, whereas the haplotypes DE1 (neutral) and DE2 (susceptible) were expressed in the roots and boles, with the exception of LG04.1g028500, which is root-specific (Figure 6c). Furthermore, overall, *G. boninense* inoculation slightly activated these three genes in haplotypes DE1 and DE3 but not in the resistant haplotype DE2 (Figure 6c).

**Figure 7:**
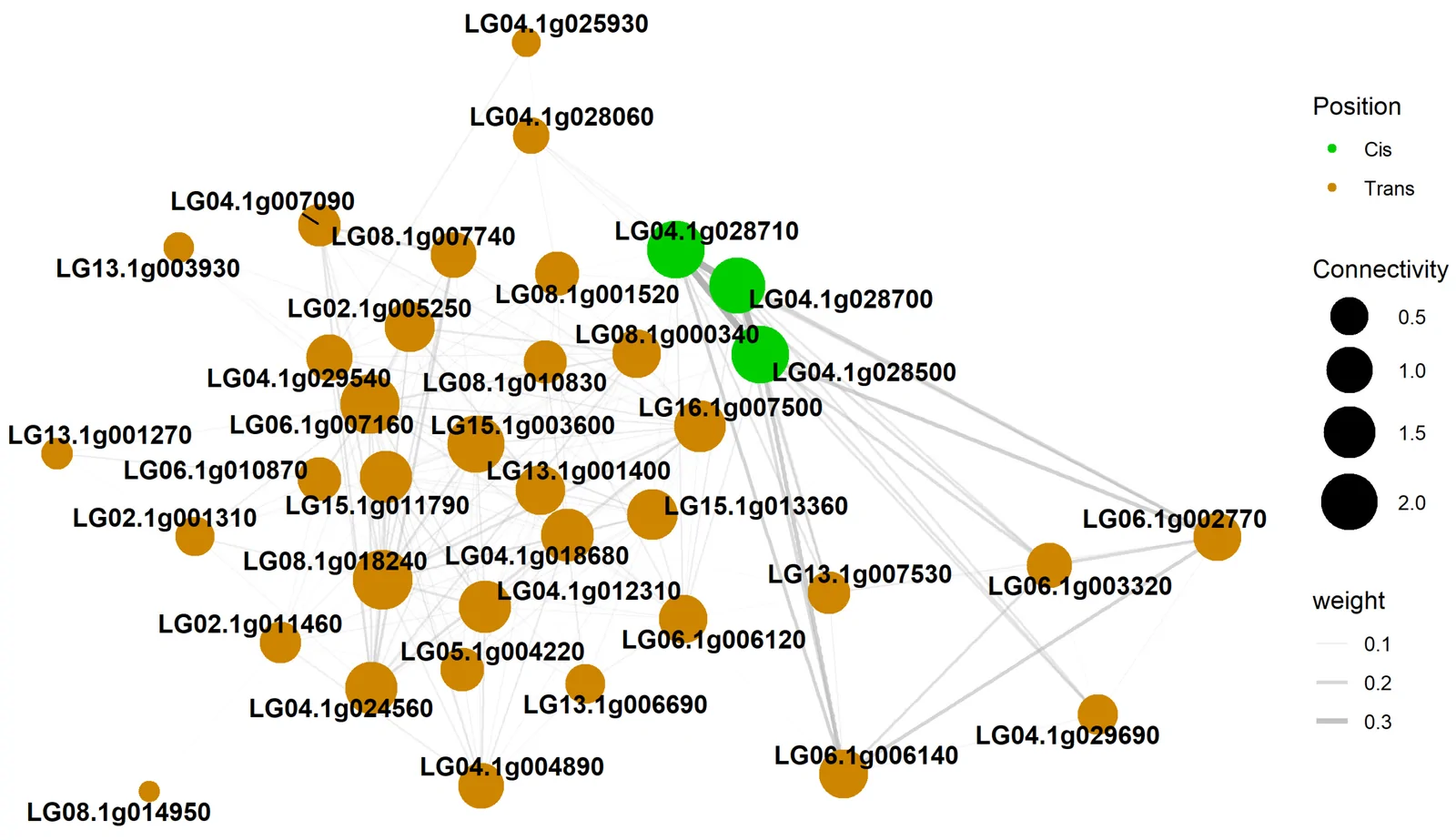
Weighted correlation network of differentially expressed genes (DEGs) relative to GA_04at177 QTL. The first module comprising 37 genes is represented, with cis-DEGs in green and trans-DEGs in orange.

In order to identify which process might be involved in the resistance conferred by the GA_04at177 QTL and to further prioritize the cis-DEGs, we performed a correlation network analysis on the list of cis- and trans-DEGs for predictors involving the effect of the GA_04at177 QTL (*n* = 113). The three genes closest to the peak and described above were the only cis-DEGs present in the first module which included 37 of the 113 DEGs (Figure 7). The three cis-DEGs were strongly connected to each other, to a lesser extent to five other genes on chromosomes 4 (LG04.1g029690), 6 (LG06.1g002770, LG06.1g003320 and LG06.1g006140), and 13 (LG13.1g007530), and had little connection to the other 29 genes (Figure 7). The three cis-DEGs with highest prioritization encode a putative albumin-2 (LG04.1g028500), an ankyrin repeat-containing protein (LG04.1g028700), and a putative disease resistance RPP8-like protein 3 (LG04.1g028710); the 5 trans-DEGs most connected to them encode an aquaporin SIP1-2 (LG04.1g029690), a branched-chain-amino-acid aminotransferase-like protein 2 (LG06.1g003320) and a transaldolase (LG13.1g007530), while two of them (LG06.1g002770, LG06.1g006140) had no functional annotation (Table S3).

## 4. Discussion

Marker-assisted selection (MAS) has become an essential tool for plant breeding. Regarding oil palm resistance to *Ganoderma boninense*, this would help overcome significant breeding challenges, namely a long selection cycle and a slowly developing disease whose resistance is governed by a complex, quantitative, and polygenic architecture [15,43,45]. The most effective approach for identifying resistance markers to be incorporated into a MAS program is typically through QTL or GWAS studies, as this method relies on genetic diversity, ideally available within the breeding program. However, for now, such studies are too scarce for oil palms, as -omics studies based on small sample sizes and low diversity are largely favored. This is likely due to biological constraints that make it difficult to establish effective experimental designs; one possible approach is to directly use the extensive data generated as part of a breeding program, albeit with significant methodological issues [37,81]. Combining QTL mapping with RNA-seq analysis is a powerful approach to characterize QTLs by prioritizing candidate genes and resistance mechanisms [82]. For perennial crops, for which classical fine-mapping approaches based on crosses or molecular methods are limited, this is particularly promising as recently demonstrated for a fungal disease affecting apples [83]. In the present study, we implemented this strategy by using genetic diversity relevant to our breeding program, while ensuring a strong connection between QTL and RNA-seq populations.

From a methodological perspective, this was made possible by two key approaches. Firstly, using Bayesian variable selection (BVS) provided a flexible framework well-suited to the constraints of our dataset, which originates from a breeding program not initially designed for genomic or genetic analyses. For the QTL mapping, BVS allowed us to simultaneously estimate and select QTL effects of both the GA and GB groups, considering specific SNPs for each group. Compared with methods implemented in other software and used in previous studies, these variable selection approaches provide greater detection power and improved accuracy [43,44]. For the identification of DEGs, BVS also offers an attractive alternative to the methods included in the standard DESeq2 package [78], as it allows for the simultaneous analysis of all explanatory variables within a single model. In our case, involving a complex design with multiple segregating QTLs and possible interactions, this enabled us to efficiently select the best model for each gene. Secondly, predicting QTL genotypes across different populations was critical, and could be achieved through the detection of identical-by-descent (IBD) genomic segments within a known pedigree [78]. Applied to disease resistance in oil palm, this approach successfully validated, under field conditions, the QTLs for BSR resistance identified in pre-nursery trials [84,85] and assessed potential trade-offs with production traits in the case of Fusarium wilt resistance QTLs [43]. In this study, leveraging the large-scale and reproducible genotyping enabled by the SNP array, we identified haplotypes using phasing and identity-by-state (IBS) information. This more flexible approach allowed us to predict QTL genotypes even in individuals without explicit pedigree links due to incomplete pedigree data or illegitimacy. As our genotyping program expands across all components of the breeding program, including genealogical populations, seed gardens and field or pre-nurseries trials, haplotype tracking will enhance the power, relevance, and consistency of results for complex BSR resistance.

Regarding the oil palm responses to *G. boninense* infection, our study confirmed the main findings reported in the literature. Enrichment analysis of the treatment effect highlighted responses to light, heat, ROS, and toxic substance stresses. The response to ROS is observed almost unanimously in studies published to date, regardless of the experimental design [25,26,35,86]. Lignin degradation by fungal lignolytic enzymes, including peroxidases and laccases, is known to generate ROS [18,87], suggesting that our experimental design corresponds to an already well-established stage of *G. boninense* colonization [26]. The enrichment of genes related to heat or high light intensity was unexpected. Nevertheless, some genes share functional annotations with oxidative stress, and genes from these pathways are known to be involved in response to *G. boninense* infection with a role as master regulators handling multiple stresses [25]. An added benefit of our approach is its focus on the mechanisms and genes involved in the response to *G. boninense* infection based on the genetic variation that may confer resistance. The polygenic nature of the resistance [15,43] is further supported by our identification of a significant number of QTLs within only two quite narrow genetic backgrounds. Moreover, the identification of multiple haplotypes segregating within genetic backgrounds, with contrasted effects, has characterized allelic series for the QTLs. For instance, the QTL on chromosome 4 comprises three haplotypes ranging from susceptible to highly resistant, highlighting the genetic complexity of BSR resistance when scaling to broader genetic diversity. However, compared to a previous SSR-based study in GB which mapped numerous loci, our denser genotyping coupled with BVS successfully revealed the genetic architecture with greater resolution. For instance, the previously identified QTL on LG13, which coincided with a genotyping gap at the end of the chromosome, was likely a ghost QTL arising from low marker density [88]; correcting such artifacts likely improved the robustness of the QTLs detected in the present study [89]. The prioritization of candidate genes within the identified QTLs will also help clarify their effects in relation to known defense mechanisms and pave the way for further molecular characterization. To achieve this, the present study integrates multiple layers of information, including the physical distance to the top SNP, haplotype-specific expression profiles in relation to resistance levels, integration into a correlation network with trans-DEGs, and finally, functional annotation. Applying this approach to a strong effect QTL in Deli, we identified three tightly linked cis-DEGs within less than 500 kb of the top SNP: a putative disease-resistance RPP8-like protein 3, an ankyrin repeat-containing protein, and a putative albumin-2.

Intriguingly, all three candidate genes share an identical expression pattern, being exclusively expressed in the susceptible haplotypes and silenced in the resistant one, suggesting a regional, haplotype-specific co-regulation mechanism. In plants, such localized co-regulation of physically neighboring genes is often mediated by chromatin folding into distinct three-dimensional structural units, such as topologically associating domains (TADs) or chromatin loops, which coordinate transcription by modulating local epigenetic states and chromatin accessibility [90]. Rather than a single causal locus, this QTL may therefore represent a co-regulated genomic cluster. To address this hypothesis, ongoing Hi-C sequencing conducted as part of the La Mé genome assembly project could help resolve the haplotype-specific 3D chromatin architecture of this locus. Furthermore, experimental evidence has already shown that such complex genetic architecture at a single locus can involve several closely linked causal genes acting synergistically, rather than a single causal gene [91,92]. Given that *G. boninense* is a hemibiotrophic pathogen with a necrotrophic phase [18], the coordinated expression of this cluster—comprising an NLR receptor (RPP8), an immune signaling regulator (ankyrin), and a metabolic modifier (albumin-2)—could trigger a cascade of cell death and metabolic shifts that would be beneficial to *G. boninense* colonization [93]. This hypothesis is supported by the presence in their most connected trans-DEGs of actors in transport and metabolic reprogramming, including an aquaporin (SIP1-2), a branched-chain-amino-acid aminotransferase-like protein 2, and a transaldolase. Conversely, the silencing of these three cis-DEGs in the resistant haplotype could provide a multi-genic loss-of-function resistance.

Our study provides valuable information on BSR resistance and a basis for integrating it into marker-assisted selection (MAS) schemes. However, the exclusion of 18 illegitimate individuals left 30 individuals, which limits the statistical power of the design (covering three tissues, under inoculated versus non-inoculated conditions) to detect differentially expressed genes (DEGs) associated with QTL effects. This is particularly challenging given the genetic architecture of this trait, which involves up to 11 QTLs, some of which exhibit multi-allelic series with up to three distinct resistance haplotypes in the RNA-seq population. With this limited sample size, the segregation of multiple QTLs also increases the risk of collinearity between QTL segregation and spurious correlations among DEG affected by QTL. While Bayesian variable selection allowed us to make the most of this dataset, future research should focus on expanding the population size to improve overall statistical power. Additionally, candidate genes identified within these regions should be validated via RT-qPCR using larger cohorts. Moreover, field validation is essential to ensure the transferability of the pre-nursery results to actual agronomic conditions. Indeed, infection timings may vary in the field, and older palms might develop mature-plant resistance mechanisms that are not expressed at the seedling stage. Furthermore, the genetic diversity of *G. boninense* populations in the field [12,13] adds another layer of complexity, which could partly explain why pre-nursery QTL effects are not always consistent in the field [43]. To date, field-based genetic studies remain scarce; to address this limitation, we have launched a large-scale genotyping program using the same SNP array as in this study across multiple field trials or seed gardens. By connecting these diverse experimental setups, this approach will allow us to validate and reinforce our QTL mapping results under field conditions. Another concern involves the potential trade-offs between BSR resistance and yield, especially given the polygenic architecture that spans multiple genomic regions. For instance, although resistance may be driven by increased lignin deposition [18,33], it remains unclear how this might affect oil-to-mesocarp ratios and overall oil yield. A negative correlation could indeed arise from colocalized QTLs governing these traits (S. Tisné, personal communication). Consequently, multi-trait analyses, particularly those incorporating yield components, are critical; a promising simulation approach based on haplotypes and their estimated effects on multiple traits is currently under development and could help address this challenge [42].

## 5. Conclusions

Our study provides a valuable resource for the scientific community, bridging the gap between genetic diversity relevant to breeding and the diversity of molecular responses to *G. boninense* infection. Combining for the first time QTL mapping and transcriptomics using powerful, consistent experimental and analytical frameworks for both, our approach successfully identified genomic regions involved in resistance and shed light on the genetic and molecular mechanisms underlying a resistance QTL. Nevertheless, further validation and deeper data mining remain necessary to identify and prioritize breeding targets. Ultimately, this work establishes a robust methodological framework to inspire future multi-omic studies, which will be essential to fully unravel the highly complex genetic architecture of oil palm resistance to *G. boninense* and support MAS strategies.

## Data Availability

Genomic sequence, gene annotation and RNA sequences are openly available in [EBI ENA database] at [https://www.ebi.ac.uk/ena/browser/view/PRJEB113782, accessed on 2 June 2026]. All results obtained at the individual gene level are provided in Table S3 (Supplementary Materials). In addition, an interactive R Shiny application developed to visualize QTL haplotype effects on gene expression is available at https://shinyapps.southgreen.fr/app/rnaseq-qtl-daval-et-al, accessed on 2 June 2026. Additional data may be obtained upon reasonable request to the corresponding author.

## Supplementary Materials

The following supporting information can be downloaded at: https://www.mdpi.com/article/10.3390/microorganisms14081712/s1, Figure S1: Pedigree of the Deli and La Mé oil palm QTL mapping populations.; Figure S2: Null distributions of posterior inclusion probabilities (PIPs) in oil palm heterotic groups A (red) and B (blue), generated via permutation tests for Bayesian variable selection QTL mapping of *G. boninense* resistance; Figure S3: Design of the RNA-seq experiment to study the transcriptomes of palm seedlings inoculated with *Ganoderma boninense*.; Figure S4: Sampling procedure for the 48 seedlings from the RNA-seq population.; Figure S5: Distribution of differentially expressed genes (DEGs) across the genome.; Table S1: Information on the QTL for *G. boninense* resistance; Table S2: Variances of genetic model components for basal stem rot resistance in oil palm; Table S3: Information on the genes surveyed in the RNA-seq analysis.

## Abbreviations

The following abbreviations are used in this manuscript:

BSR	Basal stem rot
BVS	Bayesian variable selection
CIRAD	French agricultural research and cooperation organization working for the sustainable development of tropical and Mediterranean regions
CL	Dikariotic clonal line
DE	Deli
DEG	Differentially expressed gene
GA/GB	Heterotic group A/B
IBD	Identical by descent
LM	La Mé
MAS	Marker-assisted selection
PIP	Posterior inclusion probabilities
QTL	Quantitative trait locus
RNA-seq	RNA sequencing
ROS	Reactive oxygen species
RWB	Rubber wood block
SNP	Single-nucleotide polymorphism
SSR	Simple sequence repeats
