# Supplementary material for: Early Response QTLs and DEGs Underlying Oil Palm Resistance to Ganoderma boninense in Two Breeding Populations Evaluated in Pre-Nursery Trials
Source: Microorganisms. 2026 Aug 4;14(8):1712. doi: 10.3390/microorganisms14081712 (PMC13515167; doi:10.3390/microorganisms14081712)

Figure S1: Pedigree of the Deli and La Mé oil palm QTL mapping populations. The white boxes represent individuals from the Deli (**a**) and La Mé (**b**) populations, and their names are codes used for publication. The gray boxes represent full-sib families, with the family number, "LM" or "D" to indicate the population ("LM" for La Mé and "D" for Deli), and the number of full-sibs in parentheses.

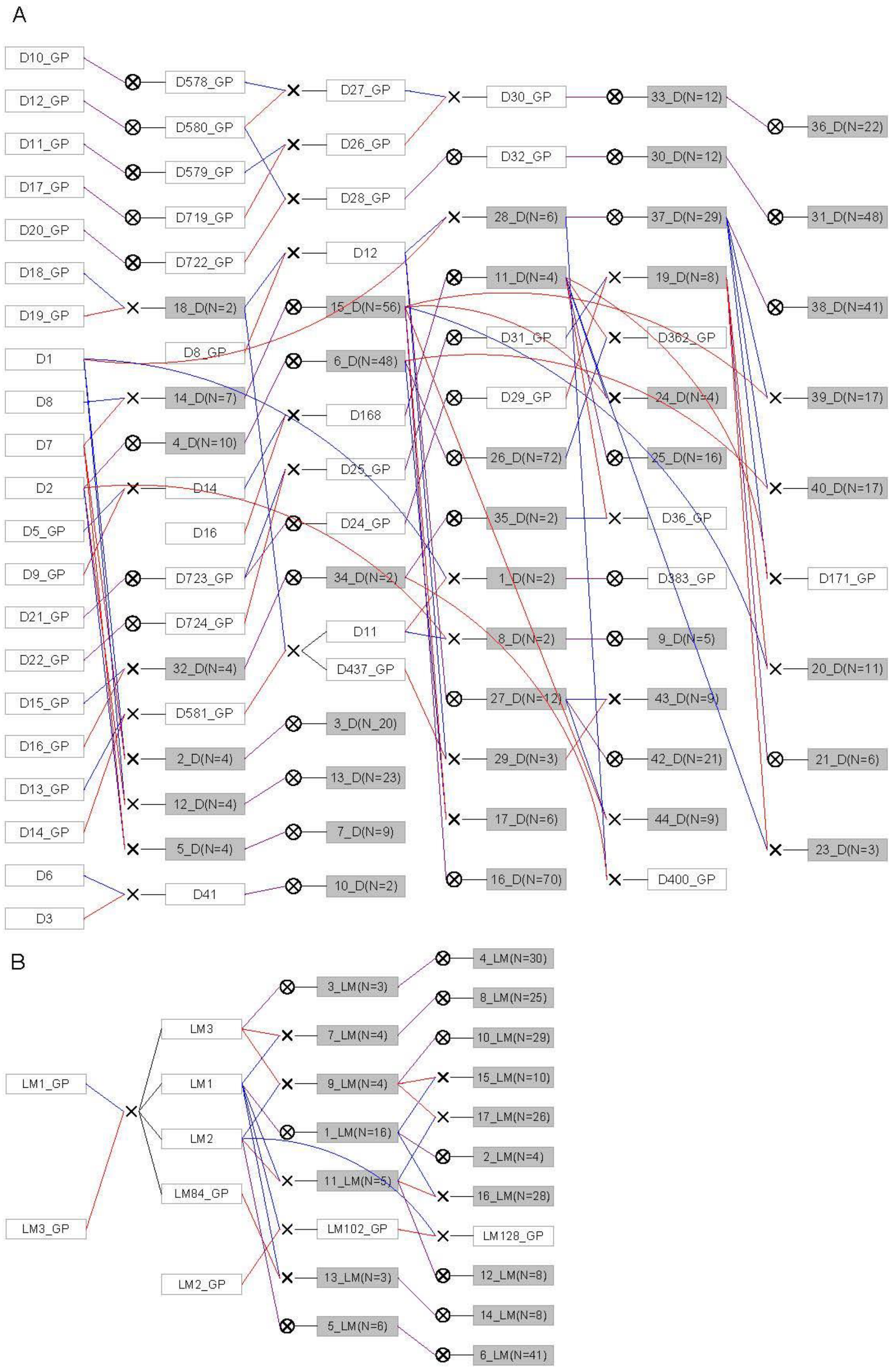

Supplement: Supplementary file 1 [file microorganisms-14-01712-s001.zip › Figure S1.pdf]
