# Supplementary material for: Early Response QTLs and DEGs Underlying Oil Palm Resistance to Ganoderma boninense in Two Breeding Populations Evaluated in Pre-Nursery Trials
Source: Microorganisms. 2026 Aug 4;14(8):1712. doi: 10.3390/microorganisms14081712 (PMC13515167; doi:10.3390/microorganisms14081712)

Figure S2: Null distributions of posterior inclusion probabilities (PIPs) in oil palm heterotic groups A (red) and B (blue), generated via permutation tests for Bayesian variable selection QTL mapping of *G. boninense* resistance.

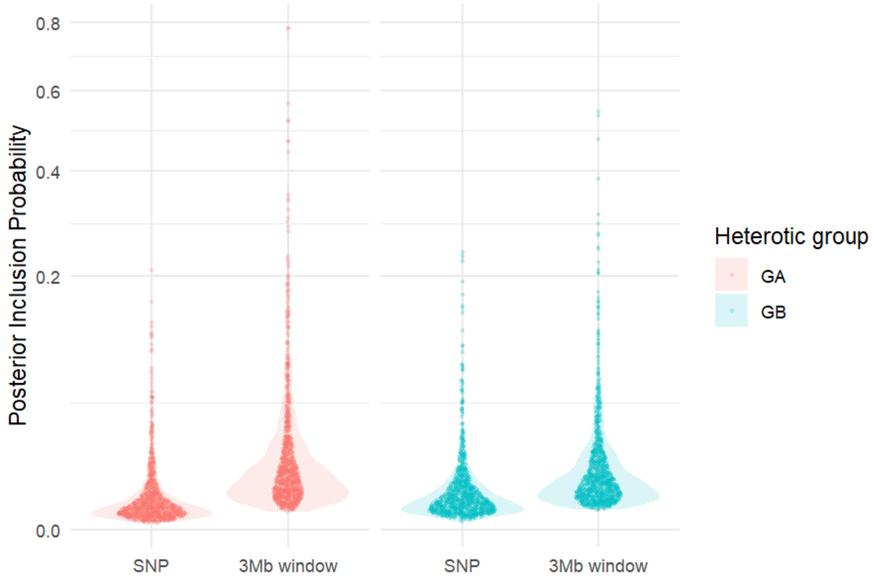

Supplement: Supplementary file 1 [file microorganisms-14-01712-s001.zip › Figure S2.pdf]
