# Supplementary material for: Early Response QTLs and DEGs Underlying Oil Palm Resistance to Ganoderma boninense in Two Breeding Populations Evaluated in Pre-Nursery Trials
Source: Microorganisms. 2026 Aug 4;14(8):1712. doi: 10.3390/microorganisms14081712 (PMC13515167; doi:10.3390/microorganisms14081712)

Figure S3. Design of the RNA-seq experiment to study the transcriptomes of palm seedlings inoculated with *Ganoderma boninense*. (**A**) Setup of the RNA-seq experiment (on the left) in addition to the routine testing trial; (**B**) Distribution of seedlings inoculated (RI) or not inoculated (RNI) with *G. boninense*, for each of the 8 crosses selected for the study; (**C**) Polybags prepared to receive an oil palm germinated seed from the selected crosses. On the right are the rubber wood blocks (RWB) inoculated with *G. boninense* and on the left control RWBs, for each cross included in the experiment.

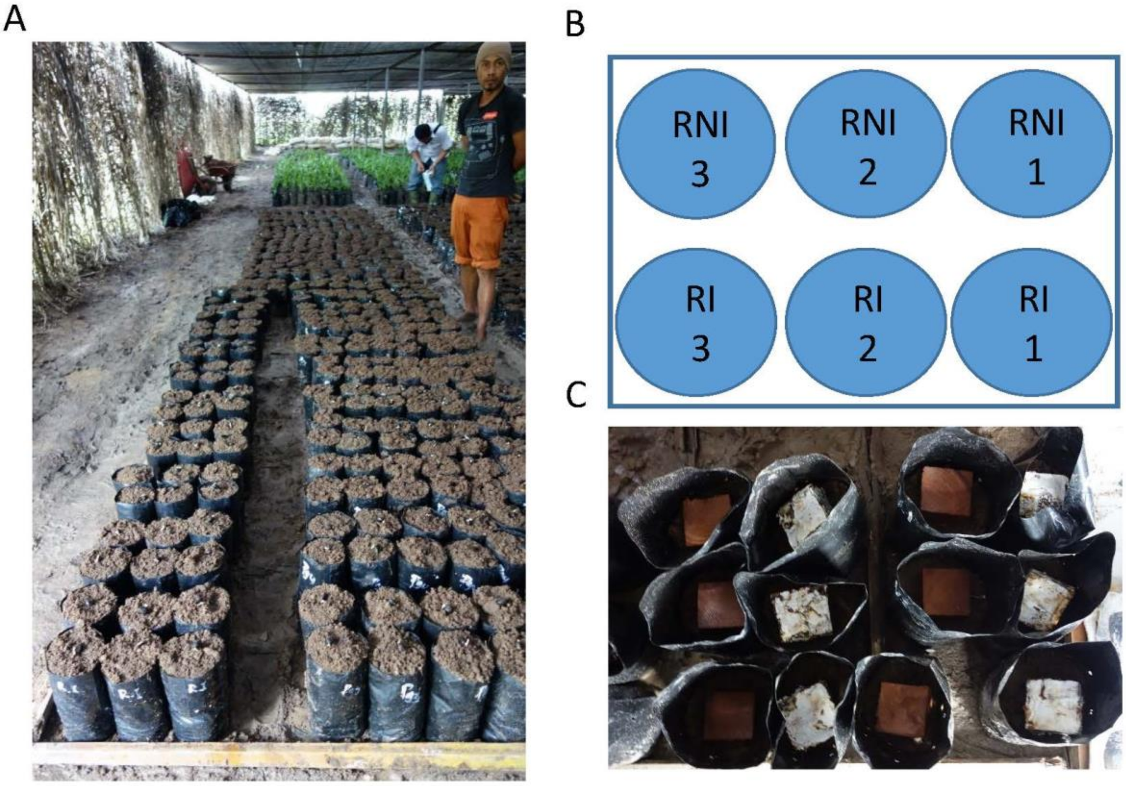

Supplement: Supplementary file 1 [file microorganisms-14-01712-s001.zip › Figure S3.pdf]
