# Supplementary material for: Early Response QTLs and DEGs Underlying Oil Palm Resistance to Ganoderma boninense in Two Breeding Populations Evaluated in Pre-Nursery Trials
Source: Microorganisms. 2026 Aug 4;14(8):1712. doi: 10.3390/microorganisms14081712 (PMC13515167; doi:10.3390/microorganisms14081712)
Supplement: Supplementary file 1 [file microorganisms-14-01712-s001.zip › Figure S4.pdf]

Figure S4. Sampling procedure for the 48 seedlings from the RNA-seq population: (**a**) The polybags are carefully opened with a scalpel, and the soil is gradually removed without damaging the roots of the seedling; (**b**) Root contact with the rubber wood block (RWB) is checked for each seedling; if there is no contact, the seedling is discarded; (**c**) Closer view of the root contact (red arrow) with the RWB, (**d**) After cleaning the roots (see Materials and Methods), the full length of the primary root's contact with the RWB is visible.

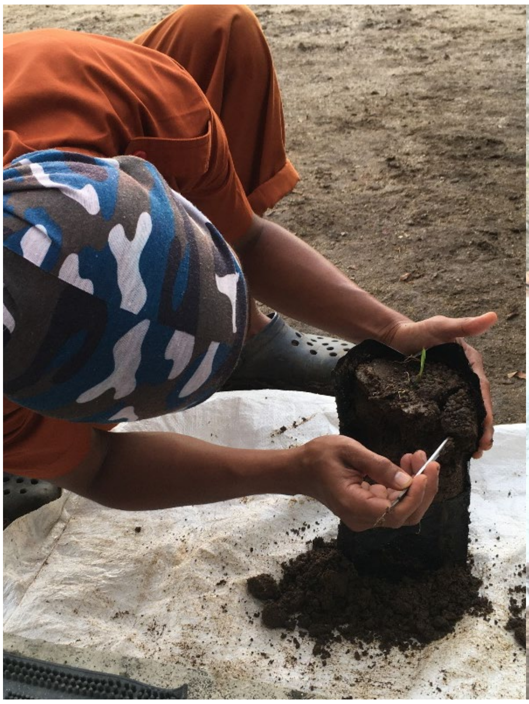

(**a**)

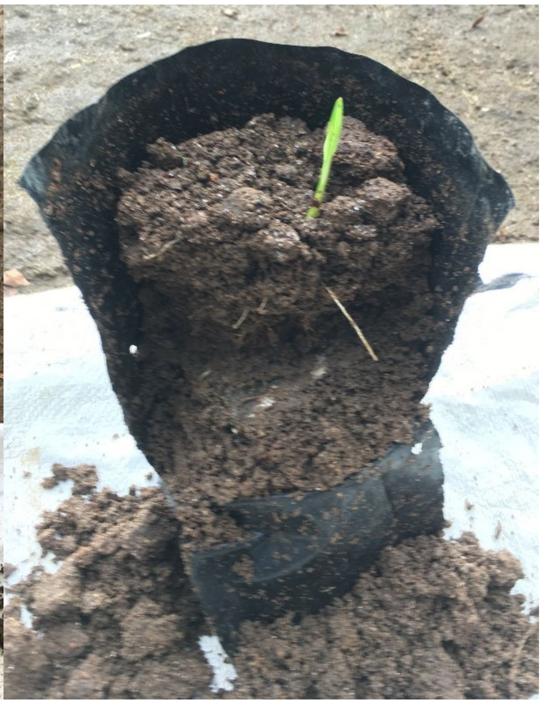

(**b**)

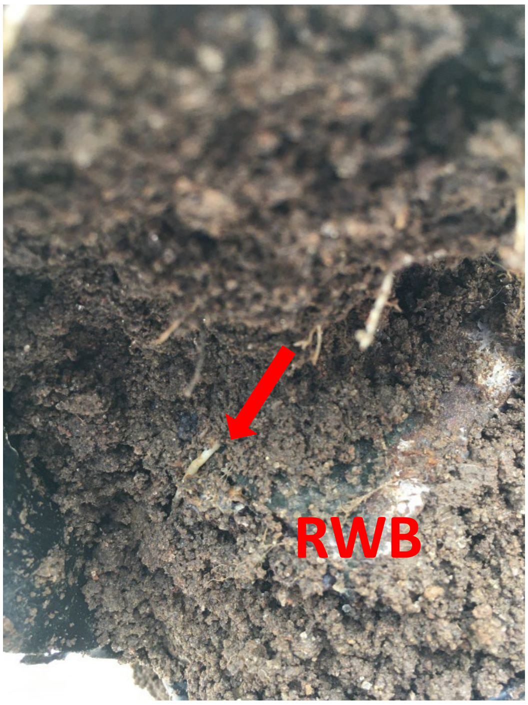

(**c**)

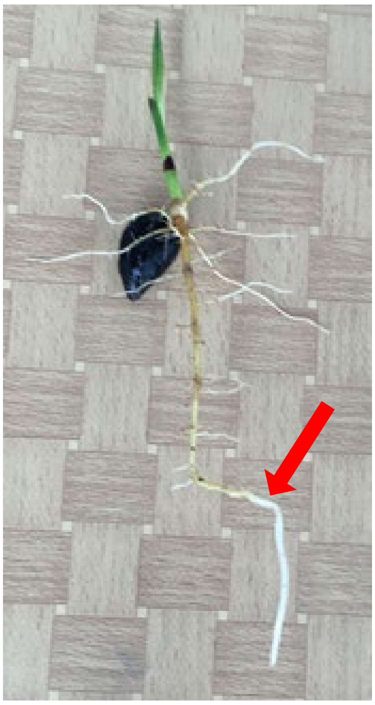

(**d**)
