# Supplementary material for: Early Response QTLs and DEGs Underlying Oil Palm Resistance to Ganoderma boninense in Two Breeding Populations Evaluated in Pre-Nursery Trials
Source: Microorganisms. 2026 Aug 4;14(8):1712. doi: 10.3390/microorganisms14081712 (PMC13515167; doi:10.3390/microorganisms14081712)

Figure S5: Distribution of differentially expressed genes (DEGs) across the genome. The number of DEGs per 1 Mb window is shown. Positions of BSR resistance QTLs are indicated by green vertical lines.

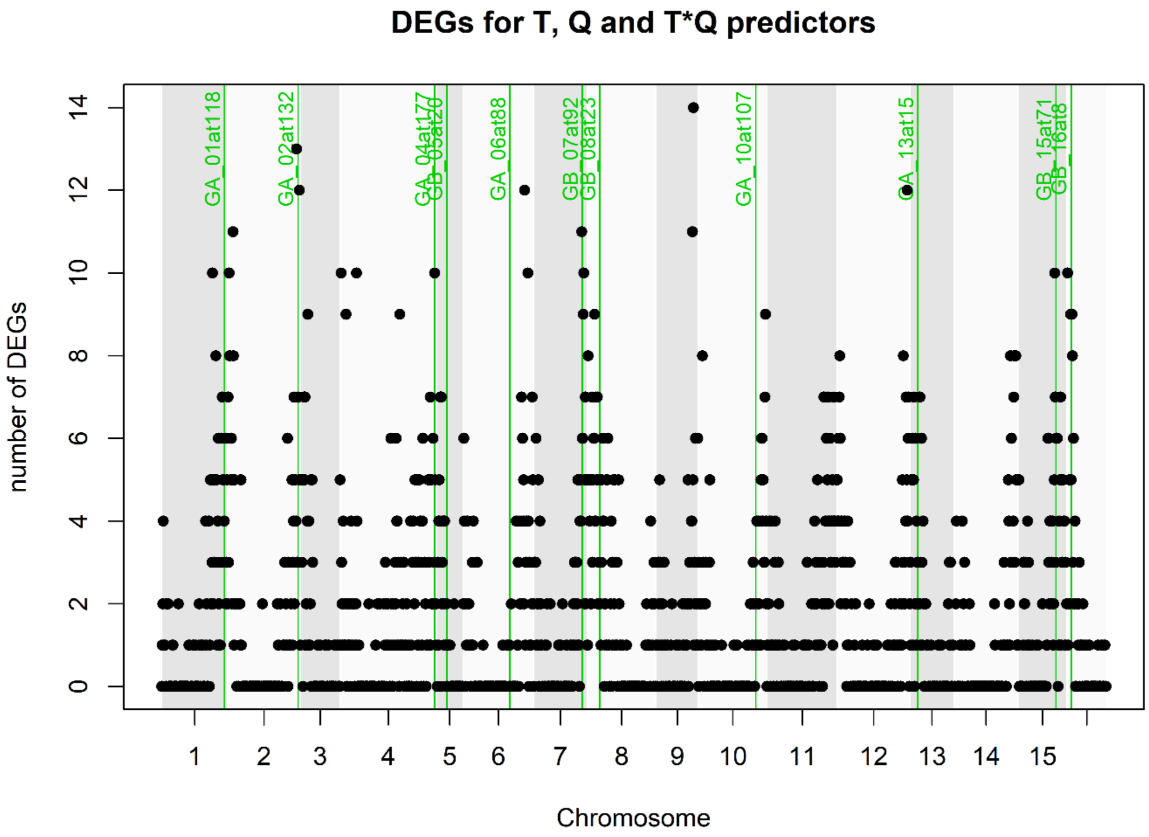

Supplement: Supplementary file 1 [file microorganisms-14-01712-s001.zip › Figure S5.pdf]
